# "What they see is what you get": Prescribing antibiotics for respiratory tract infections in primary care: Do high prescribers diagnose differently? An analysis of German routine data

**DOI:** 10.1371/journal.pone.0188521

**Published:** 2017-12-08

**Authors:** Susann Hueber, Thomas Kuehlein, Roman Gerlach, Martin Tauscher, Angela Schedlbauer

**Affiliations:** 1 Universitätsklinikum Erlangen, Institute of General Practice, Erlangen, Germany; 2 Kassenärztliche Vereinigung Bayern, München, Germany; La Trobe University, AUSTRALIA

## Abstract

**Background:**

Characteristics of high and low prescribers of antibiotics in German primary care were analysed using population data. We aimed to evaluate differences in prescribing rates and factors being associated with high prescribing, and whether high prescribers made the diagnosis of perceived bacterial infections more often.

**Methods:**

Routine data were provided by the Bavarian Association of Statutory Health Insurance Physicians. Routine data are delivered by primary care practices on a quarterly basis. We analysed data from 2011 and 2012. Patients older than 15 years with respiratory tract infections consulting a primary care physician were selected (6.647 primary care practices). Patient and physician characteristics associated with high prescribing were identified using stepwise logistic regression.

**Results:**

Mean prescribing rate of antibiotics was 24.9%. Prescribing rate for high prescribers was 43.5% compared to 8.5% for low prescribers. High prescribers made the diagnosis of perceived bacterial infections more often (*M*_high_ = 64.5%, *M*_low_ = 45.2%). In the adjusted regression model, perceived bacterial infections were strongly associated with high prescribing (OR = 13.9, 95% CI [10.2, 18.8]). Treating patients with comorbidities was associated with lower prescribing of antibiotics (OR = 0.6, 95% CI [0.4, 0.8]). High prescribers had a higher practice volume, a higher degree of prescribing dominance, and were situated more often in deprived areas and in rural settings.

**Interpretation:**

Compared to findings of studies in other European countries, prescribing rates were low. There was a considerable difference between prescribing rates of high and low prescribers. Diagnostic labelling was the best predictor for high prescribing. Current guidelines recommend considering antibiotic treatment for patients with co-morbidities. In our study, treating a large number of high-risk patients was not associated with high prescribing.

## Introduction

Antibiotics are still overprescribed for respiratory tract infections (RTI) [1]. Most RTI are of viral origin and antibiotics are rarely indicated. A recent analysis of routine data of German ambulatory care showed that antibiotics were prescribed for 30% of all patients with respiratory tract infections consulting a GP [2]. In outpatient care, clinical signs are often unspecific causing considerable diagnostic uncertainty in the differentiation between viral and bacterial infections. Due to the inevitable uncertainty of the diagnosis, guidelines recommend a more generous indication of antibiotic treatment for older patients and patients with comorbidities [3]. Clinical factors, as well as patients’ and physicians’ characteristics influence antibiotic prescribing. Poor general health seems to be associated with higher prescribing, whereas age under 60 is associated with lower prescribing [4]. Prescribing rates increase with physician’s age and time in practice [5–7], high practice volume [6, 8–10], rural practice location [11], low population density [12] and deprivation of the catchment area of the practice [13]. Prescribing rates generally differ widely between physicians, with studies suggesting a considerable influence of personal overall preference on prescribing behaviour [14–16]. Patients diagnosed with acute bronchitis were much more likely to receive an antibiotic compared to patients diagnosed with common cold [17]. Despite the fact that acute bronchitis is mainly of viral origin, it might be that the diagnosis is perceived as an illness with a potential to develop into pneumonia. Diagnoses with a potential bacterial cause such as acute sinusitis or bronchitis may serve as a false justification for antibiotic prescribing and were used by high prescribers more often [18–21]. We will call these diagnoses *perceived bacterial infections* in the sections to follow.

In our study, characteristics of high and low prescribers of antibiotics in German primary care were evaluated. Large population data derived from routine data of ambulatory care were examined. Specific objectives were: to examine how much high and low prescribers differed in their antibiotic prescribing rates, whether high prescribers made the diagnosis of perceived bacterial infections more frequently and to identify physician and patient characteristics being associated with high prescribing.

## Materials and methods

### Ethics

Approval was granted by the Ethics Committee of the Faculty of Medicine of the Friedrich-Alexander University Erlangen-Nürnberg (218_14 B).

### Claims data and criteria on data selection

To understand the circumstances around data collection, it is important to consider the characteristics of the German health care system: free provider choice and unregulated access to health care. Patients have free access to both primary care physicians (PCP) and office based specialists. Consulting more than one PCP and specialists in parallel is possible. Therefore patients can receive prescriptions from primary care physicians but also from office-based specialists in parallel. In rural areas, where there are only few specialists, most prescribing for a given patient is done by the PCP. In urban setting much of the prescribing is taken over by specialists. The so-called prescribing dominance is an indicator for the domination of prescribing activities taking place in primary care and allows evaluating to which degree a primary care practice is the main prescriber for its patients. It is defined as the proportion of prescriptions issued by a specific practice divided by all prescriptions being issued for the same patient population.

Claims data were provided by the Bavarian Association of Statutory Health Insurance Physicians (Kassenärztliche Vereinigung Bayerns, KVB). In Germany, physicians accredited with statutory health insurances send their reimbursement claims for provided ambulatory medical services to their corresponding regional KV. Data are delivered on a quarterly basis and do not contain information on a day to day basis, not allowing a direct link between prescriptions and certain diagnoses.

The provided data contained an anonymous unique patient identifier, patient’s age in five-year intervals, sex and diagnoses encoded according to the International Classification of Diseases (ICD-10-GM). Unlike the international version, the German modification of ICD-10 allows the doctors to add the strength of diagnostic reasoning such as suspected, assured, excluded or sequelae. The ICD-10 does not allow for a meaningful aggregation of its classes for our purposes. Therefore, diagnoses in the data set were transformed and grouped following the International Classification of Primary Care (ICPC 2-R, [22]), a classification system related to the World Health Organization (WHO)–International Family of Classifications. On top of being an adequate classification for the domain of primary care in itself, the ICPC-2, via an official mapping between the two classifications, allows for a meaningful aggregation based on ICD-10 into ICPC-2 codes.

The data set revealed details about physicians’ age (in five-year intervals) and sex, practice location (rural, urban, large city and administrative district), practice type (single-handed or group practice, number of physicians per surgery) and specialist training (trained GP, specialist in internal medicine working as primary care physician or physician without specialist training; hereinafter all of them together called primary care physicians (PCP)). Further information was provided on practice volume per quarter, regional deprivation coded as Bavarian Index of Multiple Deprivation (BIMD, [23]) and on degree of prescribing dominance. Patients’ and physicians’ data were linked to all prescriptions issued by a specific surgery. The origin of prescriptions cannot be identified beyond the practice level. In case of group practices, linking prescription data to PCPs’ individual data, such as age or sex, is not possible. Details on medication are encoded following the Anatomical Therapeutic Chemical (ATC) classification [24].

Data on physicians and patients were anonymized. German law allows for analysing anonymized patient data for scientific issues without formal consent of the patients. All relevant data protection requirements were taken into consideration. Data captured in 2011 and 2012 were analysed (eight accounting quarters). The following data were provided: (1) Patients with at least one of the following ICPC-diagnoses in one quarter: R72 (strep throat), R74 (acute upper respiratory infection), R75 (acute/chronic sinusitis), R76 (acute tonsillitis), R77 (acute laryngitis/tracheitis), R78 (acute bronchitis/bronchiolitis), R80 (influenza), R81 (pneumonia) and/or (2) patients who received a prescription of antibiotics (ATC J01) and/or of neuraminidase inhibitors (J05 AH).

### Data processing and filtering

**Fig 1:**
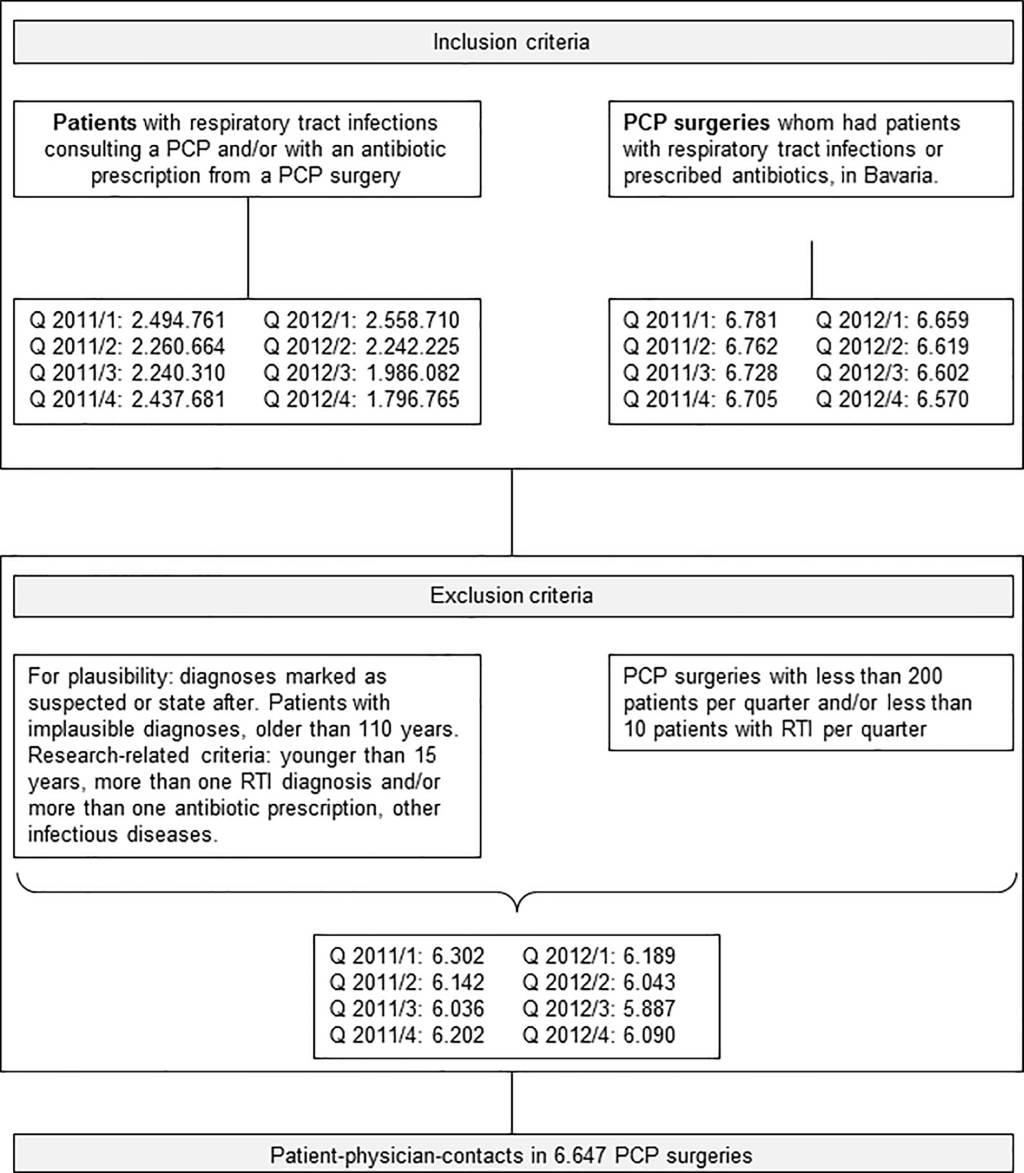
Flowchart for data selection and filtering and to illustrate characteristics of PCPs and patients.

Data preparation and selection process is depicted in Fig 1. Patients with a diagnosis of RTI as defined above who had seen a PCP in Bavaria were selected. Only patients with one diagnosis of a RTI and/or one antibiotic prescription per quarter were included in order to allow an association between diagnosis and prescription. Exclusion criteria were: Patients with incompatible or implausible coding combinations, younger than fifteen years and/or with a diagnosis of other infectious diseases. Comorbidity was determined using the Charlson comorbidity index [25]. Primary care practices with less than 200 patients per doctor per quarter and with less than ten patients with RTIs per doctor per quarter were excluded in order to select “typical” primary care providers only. The following antibiotics used in the treatment of RTIs were selected through their ATC-codes: tetracycline (J01A), beta-lactam antibiotics penicillin (J01C), other beta-lactam antibacterial like first-, second-, third- and fourth generation cephalosporin (J01DB, J01DC, J01DD, J01DE, respectively) and other cephalosporin (J01DI), macrolides, (J01F) and quinolones (J01M). Data of patient-physician-contacts in 6.647 PCP practices were analysed.

### Data analysis

Antibiotic prescribing rate per practice was defined as the proportion of patients with an antibiotic prescription divided by the number of patients with RTI. Data of all eight quarters have been summarized. PCP sample was stratified for prescribing rate into low prescribers (<25th percentile) and high prescribers (>75th percentile) [16]. Diagnostic labelling was defined as the proportion of a specific RTI diagnosis (numerator) and the sum of all RTI diagnoses (denominator). Also, the proportion of the sum of all perceived bacterial infections of all RTI diagnoses was calculated. The following diagnoses were counted as perceived bacterial infections: R72 (strep throat), R75 (acute/chronic sinusitis), R76 (acute tonsillitis), R77 (acute laryngitis/tracheitis), R78 (acute bronchitis/bronchiolitis), and R81 (pneumonia). Differences between types of prescriber were tested statistically applying Student’s t-test with a significance level at 5%. Effect sizes using Cohen’s d were calculated [26].

Factors associated with type of prescriber were examined using stepwise binary logistic regression, the depending variable being the type of prescriber (high vs. low). As we were interested to contrast factors associated with high prescribing behaviour as compared to factors associated with low prescribing, only data of high prescribers and low prescribers practices were included in the analysis. As described above, data were stratified for prescriber type and therefore we included data of 1.662 high prescriber practices and data of 1.662 low prescriber practices (in sum N = 3.324 primary care practices). Predictors were: (1) patient characteristics: proportion of patients with perceived bacterial infections, proportion of patients older than 65 years and proportion of patients with co-morbidities (indicated by the proportion of patients with a Charlson index > 0), (2) practice characteristics: practice volume per quarter, index of regional deprivation (BIMD, [22]), prescribing dominance, type of practice (single-handed vs. group practice) and practice location (rural vs. urban/large cities). All predictors, except type of primary care practice and practice location, were continuous variables. To simplify the interpretation of odds ratios in the regression model, all continuous variables were transformed in categorical variables using quintiles. For each categorical variable, the lowest quintile acted as the reference category. Crude and adjusted odds ratios (OR) were determined. In the adjusted model all variables were taken into account. OR described the degree of increasing or decreasing odds of high prescribers in a specific quintile category as compared to the odds of high prescribers in the lowest quintile. As physicians’ age and gender could only be linked to prescription data on practice level, these analyses were restricted to single-handed practices. To analyse the effect of physicians’ age, Mann-Whitney-U-Test was applied and effect size was calculated (formula according to Cohen [26]: r = Z / sqrt (N)). To evaluate, whether gender has an effect, a Chi-squared test was applied.

## Results

Antibiotic prescribing rate: The mean prescribing rate was *M* = 24.9% (*SD* = 14%). High prescribers had a prescribing rate above 33.9%, whereas low prescribers were below 14.9%. Mean prescribing rate within the high prescriber group was *M*_high_ = 43.5% (*SD* = 7.7%) compared to *M*_low_ = 8.5% within the low prescriber group (*SD* = 4.5%, *t*(2681.34) = -158.93, *p* < .01, *d* = 5.63).

Diagnostic labelling: Results and statistical analysis can be seen in Table 1. The most frequent diagnosis was acute upper respiratory tract infection (R74; *M* = 44.5%) followed by acute bronchitis (R78; *M* = 31.9%). Low prescribers diagnosed significantly more often acute upper respiratory tract infections (*M*_low_ = 52.4% vs. *M*_high_ = 33.9%; *t*(3278.3) = 23.3, *p* < .01, *d* = 0.8), whereas acute bronchitis was diagnosed more often by high prescribers (*M*_low_ = 25.2% vs. *M*_high_ = 41.1%; *t*(3292.8) = -23.7, *p* < .01, *d* = 0.8). Over all practices, the mean proportion of perceived bacterial diagnoses was *M* = 53.3% (*SD* = 22.9%). Perceived bacterial infections were diagnosed more often by high prescribers (*M*_high_ = 64.5%, *M*_low_ = 45.2%, *t*(3299.9) = -24.87, *p* < .01, *d* = 0.9). Effect sizes indicate that differences are of clinical relevance.

**Table 1:** Diagnostic labelling. Proportion of a specific RTI diagnosis on the sum all RTI diagnoses per practice. Mean values over all practices are shown in column 2. Mean values separately for high prescribers and low prescribers are shown column 3 and 4 respectively. Results of inferential statistical analyses can be seen in column 5.

	Proportion of patients with a specific diagnoses			
	All surgeries	High prescribers	Low prescribers	High vs. low prescribers
	(N = 6.647)	(N = 1.662)	(N = 1.662)	
	*M* (*SD*)	*M* (*SD*)	*M* (*SD*)	*t*, *p*-value, 95% CI, Cohen’s *d*
R72 Strep Throat	0.5% (1.7%)	0.6% (2.2%)	0.4% (1.2%)	*t*(2604.3) = - 3.7, *p* < .001, 95% CI [-.4; -.1], d = 0.1
R74 Upper respiratory infection, acute	44.5% (23.2%)	33.9% (21.4%)	52.4% (24.1%)	*t*(3278.3) = 23.3, *p* < .001, 95% CI [16.9; 19.9], *d* = 0.8
R75 Sinusitis acute / chronic	15.5% (10.9%)	16,.5% (10.8%)	15.6% (11.3%)	*t*(3254.5) = - 3.5, *p* < .001, 95% CI [-2.3; -.7], *d* = 0.1
R76 Tonsillitis acute	0.07% (0.2%)	0.08% (0.2%)	0.06% (0.2%)	*t*(3265.2) = - 2.4, *p* = .01, 95% CI [-.04; -0.01], *d* = 0.1
R77 Laryngitis / Tracheitis acute	2.9% (5.2%)	3.7% (7.1%)	2.3% (3.5%)	*t*(2401.4) = -1.3, *p* < .001, 95% CI [-1.8; -1.0], *d* = 0.3
R78 Acute bronchitis / bronchiolitis	31.9% (19.2%)	41.1% (20.1%)	25.2% (18.3%)	*t*(3292.8) = - 23.6, *p* < .001, 95% CI [-17.1; -14.5], *d* = 0.8
R80 Influenza	2.2% (7.7%)	1.5% (5.4%)	2.4% (8.6%)	*t*(2792.5) = - 3.4, *p* < .001, 95% CI [0.4; 1.4], *d* = 0.1
R81 Pneumonia	2.4% (4.2%)	2.4% (5.3%)	2.1% (3.8%)	*t*(3322.0) = 2.2, *p* = .03, 95% CI [-.7; -.1], *d* = 0.1
Perceived bacterial infections	53.3% (22.9%)	64.5% (21.4%)	45.2% (23.3%)	*t*(3299.9) = - 24.8, *p* < .001, 95% CI [-20.8; -17.8], *d* = 0.9

Factors associated with high prescribing: The regression analysis model can be seen in Table 2. Crude OR indicated a strong association between high prescribing and diagnostic labelling, higher age of patients and comorbidities. Crude odds for being a high prescriber were eleven times higher in practices with the highest rates of perceived bacterial infections compared to practices with the lowest rates (crude OR for highest quintile = 10.8, 95% CI [8.5,13.8]. In the fully adjusted model, adjusted OR for patients’ characteristics changed. Adjusted odds for high prescribers increased to being 14 times higher in practices with the highest proportion of perceived bacterial infections (adjusted OR = 13.9, 95% CI [10.2, 18.8]). Interestingly, the OR for practices with the largest number of patients with comorbidities decreased to 0.6 when being adjusted for other factors in the model (95% CI [0.4, 0.8]. This means, a 40% decrease of high prescribers in practices with the largest number patients with comorbidities compared to practices with the lowest number of patients with comorbidities. A high proportion of patients at old age was not associated with high prescribing (adjusted OR = 0.9, 95% CI [0.7, 1.2]). In the adjusted model, structural factors such as higher practice volume, deprived area, higher prescribing dominance and practice in rural area remained having a strong positive association with high prescribing. Being a physician in a single-handed practice strengthened the negative association with high prescribing.

**Table 2:** Logistic regression model to analyse factors associated with type of prescriber (high vs. low). Quintiles of continuous variables are shown in column 2. Crude OR are shown in column 3 and adjusted OR including all variables can be seen in column 4.

	Quintile	Crude OR	95% CI	p	Adjusted OR	95% CI	p
Prop. of patients with perceived bacterial infections	< P_20_ = 33%			< .001			< .001
	- P_40_ = 45%	2.1	[1.6, 2.8]	< .001	2.4	[1.8, 3.2]	< .001
	- P_60_ = 59%	4.1	[3.2, 5.3]	< .001	4.8	[3.6, 6.4]	< .001
	- P_80_ = 75%	8.8	[6.9, 11.2]	< .001	10.3	[7.6, 11.3]	< .001
	> P_80_	10.8	[8.5, 13.8]	< .001	13.9	[10.2, 18.8]	< .001
Prop. of patients with RTI > 65 years	< P_20_ = 13%			< .001			< .001
	- P_40_ = 17%	2.2	[1.8, 2.8]	< .001	1.5	[1.1, 1.9]	= .007
	- P_60_ = 21%	2.4	[1.9, 3.0]	< .001	1.3	[0.9, 1.7]	= .104
	- P_80_ = 28%	3.1	[2.5, 3.8]	< .001	1.3	[0.9, 1.7]	= .065
	> P_80_	2.3	[1.9, 2.9]	< .001	0.9	[0.7, 1.2]	= .426
Prop. of patients with comorbidities	< P_20_ = 17%			< .001			< .001
(Charlson-Score > 1)	- P_40_ = 24%	1.3	[1.0, 1.6]	= .02	0.8	[0.6, 1.1]	= .149
	- P_60_ = 32%	1.4	[1.2, 1.8]	< .001	0.6	[0.5, 0.8]	= .002
	- P_80_ = 44%	1.8	[1.5, 2.3]	< .001	0.6	[0.5, 0.8]	< .001
	> P_80_	2.9	[2.4, 3.7]	< .001	0.6	[0.4, 0.8]	< .001
Practice volume per quarter	< P_20_ = 545			< .001			< .001
	- P_40_ = 724	1.9	[1.6, 2.5]	< .001	1.6	[1.2, 2.0]	< .001
	- P_60_ = 906	2.6	[2.1, 3.3]	< .001	2.0	[1.5, 2.6]	< .001
	- P_80_ = 1134	3.4	[2.6, 4.2]	< .001	2.5	[1.9, 3.3]	< .001
	> P_80_	5.9	[4.7, 7.5]	< .001	3.8	[2.9, 5.0]	< .001
Prescribing dominance	< P_20_ = 40%			< .001			< .001
	- P_40_ = 48%	2.5	[2.0, 3.2]	< .001	2.4	[1.8, 3.0]	< .001
	- P_60_ = 54%	3.4	[2.7, 4.3]	< .001	2.4	[1.8, 3.1]	< .001
	- P_80_ = 61%	3.9	[3.1, 4.9]	< .001	2.8	[2.0, 3.5	< .001
	> P_80_	5.1	[4.0, 6.4]	< .001	3.6	[2.6, 4.5]	< .001
Regional Deprivation, BIMD	< P_20_ = 11.36			< .001			< .001
	- P_40_ = 16.46	1.1	[0.9, 1.3]	= .454	1.7	[1.3, 2.2]	< .001
	- P_60_ = 22.68	2.0	[1.6, 2.5]	< .001	1.8	[1.4, 2.3]	< .001
	- P_80_ = 31.58	3.2	[2.5, 4.0]	< .001	3.3	[2.5, 4.4]	< .001
	> P_80_	2.8	[2.2, 3.4]	< .001	4.6	[3.3, 6.2]	< .001
Rural practice		1.9	[1.7, 2.2]	< .001	2.0	[1.6, 2.5]	< .001
Single-handed practice		0.8	[0.6, 0.9]	< .001	0.7	[0.6, 0.8]	< .001
Constant					0.02		

Regarding physicians’ age and gender, the difference between high prescribers and low prescribers was analysed for single-handed practices only. Concerning age, the difference was significant (*Median*_*low*_ = 57.8 years, *Median*_*high*_ = 60.0 years, *p* < 0.02), but an effect size of *r* = 0.03 indicated that the difference is negligible. The analysis of gender showed that women were more likely to be in the low prescriber group (women: low prescriber: 56.2%, high prescriber: 43.8%, men: low prescriber: 50.3%, high prescriber: 49.7%, *Χ* (1) = 6.4, *p* = .01).

## Discussion

Across primary care practices in Bavaria antibiotics were prescribed for roughly a quarter of patients with respiratory tract infections. Prescribing rates differed considerably between physicians: high prescribers were five times more likely to prescribe antibiotics. High prescribers diagnosed more perceived bacterial infections, this in turn being the best predictor for high prescriber type. Being a physician working in a practice with a multimorbid practice population was the best predictor for low prescribing. High and low prescriber practices differed in structural factors: high prescribers had a higher practice volume and a higher degree of prescribing dominance. They were more likely to work in deprived areas and in rural settings.

Compared to our study, a similar prescribing rate was found in an earlier routine data analysis of German ambulatory care (Bavaria: 27%, [27]). Clearly higher prescribing rates were found in studies conducted in the Netherlands, the United Kingdom and Sweden [1, 28–31]. There are two possible explanations. Firstly, the finding could be attributed to methodological differences. We applied restricted inclusion criteria to allow for an association between diagnosis and prescription, e.g. patients with other infectious diseases and with more than one diagnosis of RTI were excluded. Eliminating patients with a more severe clinical course could have resulted in a diluting effect and an underestimation of true prescribing rates. Secondly, differences in health care systems are possible reasons for inconsistencies in prescribing rates. In the UK and in Scandinavian countries with strictly implemented primary care systems, GPs account for nearly all prescriptions for an individual patient, whereas in Germany patients also receive prescriptions from office-based specialists. We assume that co-prescribing by specialists was leading to lower prescribing rates for PCPs with a lower prescribing dominance in our study. This is supported by the fact that higher prescribing rates were associated with a higher degree of prescribing dominance (see Table 2).

Our results support previous findings of considerable differences in prescribing rates between physicians [14, 15, 30, 32, 33]. The structural factors of practices we identified were consistent with those found in other studies: higher practice volume [6, 9, 10], regional deprivation [13, 33] and rural setting [11] are associated with high prescribing. The strongest association with high prescriber type was found for diagnostic labelling. This also confirms results found in earlier studies [18, 19, 21, 34]. In a German study, when asked about reasons to prescribe antibiotics, physicians reported that patient-related factors such as age and comorbidities had a strong influence on their decision [35]. Surprisingly, in our study comorbidity was associated with lower prescribing. Looking at patient factors such as age or comorbidity alone, the analysis showed that these factors are associated with high antibiotic prescribing rates (crude OR in highest quintiles = 2.3 and 2.9, respectively; see Table 2). This association disappeared when adjusting for other relevant factors such as diagnostic labelling (adjusted OR in highest quintiles 0.9 and 0.6, respectively; see Table 2). This aspect of our findings was confirmed by other studies in the medical literature. An analysis of medical records by Aspinall *et al*. confirmed that both a diagnosis of acute bronchitis and comorbidity were associated with high antibiotic prescribing [7]. However, there was a much weaker association to comorbidity [7]. An evaluation of high and low prescribing in another study found that patients with low comorbidity were equally covered by both low and high prescribers [36]. Sutter *et al*. concluded that whether or not a patient received an antibiotic was mainly determined by physicians’ personal attitude and not so much by the clinical picture, and that the tendency to prescribe medication in general and a defensive attitude were related to antibiotic prescribing [14]. Other studies found that a diagnosis might serve as a justification for treatment choice [18, 19]. Patients with acute respiratory tract infections often show nonspecific symptoms making a valid diagnosis more difficult. So one might ask whether in this case diagnostic labelling is closely related to prescribers’ personal traits. With our data, we cannot prove the assumption that prescribing behaviour and diagnostic labelling are related to personal characteristics. Due to the limitations of secondary data, further research using primary data or data linkage of primary and secondary data should aim to determine the causal relationship between prescribing, diagnostic labelling and patient characteristics. This could be achieved by applying a study design linking routine data to questionnaire studies in order to investigate the role of personal attitudes such as tolerance of ambiguity [37, 38].

### Limitations

The limitations of secondary data are well-known and mainly caused by the fact that the original purpose to capture those data was for billing purposes, not for research. This raises the question of data accuracy, precision and completeness. Inclusion and exclusion criteria were restricted to patients with one diagnosis and/or one antibiotic prescription to indirectly make probable a direct link between diagnosis and prescription. As a result, generalisability of our results may be somewhat reduced.

### Conclusion

The rates of antibiotic prescribing were relatively low compared to the UK or to Scandinavian countries. There was a considerable difference between prescribing rates of high and low prescribers. Diagnostic labelling was the best predictor for high prescribing. Structural factors of primary care practices were also strong influential factors. In contrast to what we had expected, patient comorbidity was not associated with high prescribing rates, when adjusted for other factors.
